# Women at Greater Sexual Risk for STIs/HIV Have a Lower Mesolimbic and Affective Bias Response to Sexual Stimuli

**DOI:** 10.3389/fnbeh.2019.00279

**Published:** 2020-01-10

**Authors:** Paul S. Regier, Anne M. Teitelman, Kanchana Jagannathan, Zachary A. Monge, Calumina McCondochie, Jaclynn Elkind, Anna Rose Childress

**Affiliations:** ^1^Department of Psychiatry, Perelman School of Medicine, University of Pennsylvania, Philadelphia, PA, United States; ^2^School of Nursing, University of Pennsylvania, Philadelphia, PA, United States

**Keywords:** risk-taking, women, fMRI, STIs, HIV, condoms

## Abstract

Young adult women in the United States have high rates of sexually transmitted infections, increasing the risk of human immunodeficiency virus (HIV). The underlying neurobiology of behaviors that increase the probability of contracting sexually-transmitted diseases (STIs) and HIV is just beginning to be explored. The current study assessed the link between sexual risk and the brain and behavioral response to sexual cues in emerging adult women. Our hypothesis was that women with more activity in reward/motivational circuitry would report higher sexual risk behaviors and would evidence higher positive affective bias to visual sexual stimuli. Women (*n* = 52; age = 18–24 years) who had protected sex 100% of the time (*n* = 17) vs. those who did not (*n* = 35), in the past 3 months, were compared on their brain response to 500 ms evocative (sex, aversive, food) vs. neutral cues in a blood-oxygen-level-dependent (BOLD) functional magnetic resonance imaging (fMRI) fast event-related design. Based on existing literature, an *a priori* anatomical “cue-reactive” mask was used to constrain the analyses. Self-reported sexual activity and the affective bias scores to sexual cues were examined as correlates with the brain response to cues. In contrast to our initial hypothesis, the higher sexual risk (Unprotected) group had significantly *less* activation in mesolimbic brain regions and lower (less positive) affective bias scores to sexual cues compared to the lower risk (Protected) group. As predicted, the brain response was positively correlated with sexual bias. Follow-up analyses showed an effect of partner “risk” (e.g., more vs. less knowledge of partner’s STIs/HIV status). This evidence suggests that women who have protected sex may view sexual-related stimuli more positively, reflected by a neural response in reward/motivational regions and more positive sexual bias scores. In contrast, young women at increased risk for STIs/HIV may feel more negatively about sexual-related stimuli, evidenced by a lower mesolimbic response and a less positive affective bias to sexual cues. These data may help identify young women who are at greatest risk for acquiring STIs and/or HIV, which carries added importance with the availability of new medications that can prevent HIV.

## Introduction

Rates of sexually-transmitted infections (STIs) have been on the rise since the early 2010s. In 2017, the Center for Disease Control reported a 22% increase in chlamydia infections, a 67% increase in gonorrhea, and a 76% increase in syphilis (Centers for Disease Control and Prevention, 2017). Individuals in late adolescence and emerging adulthood, whose regulatory brain regions are still in development, are particularly at risk, with 50% of STIs occurring in these age groups (Centers for Disease Control and Prevention, 2017). Women aged 20–24 years had the highest rate of reported chlamydia cases compared with any other age group, and rates of gonorrhea among women aged 15–24 years was higher than in men of the same age group (Centers for Disease Control and Prevention, 2017). In addition to adverse health outcomes (such as pelvic inflammatory disease and ectopic pregnancy), women with STIs are also at increased risk of contracting human immunodeficiency virus (HIV; Centers for Disease Control and Prevention, 2018a). Although the overall rate of new HIV infections has decreased in the United States over the past decade, the epidemic persists.

For women, who made up 19% of new HIV diagnoses in 2017, 87% of which were due to heterosexual contact (Centers for Disease Control and Prevention, 2018b), condoms can effectively prevent new infections. However, the use of a condom requires participation from a male partner, and this process of negotiation (Pulerwitz et al., 2002) may be especially challenging during the period of adolescence and emerging adulthood (Teitelman et al., 2011) when the brain is still developing (Sowell et al., 2004; Casey et al., 2008). For example, motivational circuits that encode reward may receive considerably less oversight from still-developing inhibitory brain regions (Ernst et al., 2005; Steinberg, 2005; Eshel et al., 2007; Casey et al., 2008; van Duijvenvoorde et al., 2010). Thus, investigating the motivational circuits that underlie behaviors that increase risk of STIs/HIV may help to identify vulnerable phenotypes and lead to interventions.

Research has begun to reveal neural correlates associated with behavior that increases the risk of STIs, much of which has focused on adolescents and the role of regulatory circuits. These circuits allow an individual to evaluate choices and future consequences associated with a particular behavior (e.g., whether to have sex or not) and enable inhibition of behavior associated with risks (e.g., sex without a condom, Miller, 2000; Bechara and Van Der Linden, 2005; Ghazizadeh et al., 2012). Studies have shown, for example, that activation in the dorsolateral prefrontal cortex and other regulatory regions during inhibition of perseverative responses and cognitive interference is correlated with more sexual risk behaviors (Feldstein Ewing et al., 2015; Barkley-Levenson et al., 2018; Hansen et al., 2018; but see Goldenberg et al., 2013), with researchers suggesting a greater potential compensatory regulatory action to inhibit prepotent responses (Hansen et al., 2018), presumably driven by hyperactive reward and emotional brain regions.

Emerging research is investigating reward-processing motivational circuits as neural correlates of sexual risk behaviors. Stimuli associated with reward act as powerful incentives for individuals to make decisions that lead to rewarding goals and previous literature has suggested that sexual cues presented in a laboratory setting can act as rewards (Gola et al., 2016). Thus, probing motivational and reward circuits with evocative stimuli, such as sexual images, may reveal differences in brain response associated with sexual risk. Prior studies suggest a heightened response in striatal and other mesolimbic regions to sexual stimuli is associated with greater sexual risk (Seok and Sohn, 2015) and compulsive sexual (Voon et al., 2014) behaviors in males. However, to our knowledge, very few previous studies have investigated the brain response to sexual stimuli as it relates to sexual risk behaviors in females. One study in females found that a heightened reward response to sexual images was associated with future sexual desire (Demos et al., 2012), though sexual risk behaviors *per se* were not investigated. Based on previous findings showing a relationship of increased mesolimbic response to greater sexual risk in males, in addition to studies generally suggesting sensitivity to cues is associated with higher risk behavior (Flagel et al., 2009; Morrow et al., 2011), we hypothesized that activation of mesolimbic regions to sexual cues would be associated with higher sexual risk in emerging adult women. Worth noting, though the direction of effects in women may differ from men, they would be important to characterize.

Passive viewing of explicit sexual stimuli can elicit feelings of embarrassment and/or shame, potentially complicating interpretation of the neural response. However, *implicit* measures of affective bias can provide a greater understanding of the brain’s response to evocative visual stimuli, without the confounds of embarrassment and/or shame. Previous research has shown that affective bias can aid in understanding more automatic decision-making (e.g., classical conditioning), such as approach or avoidance behaviors triggered with little or no conscious thought (Olson and Fazio, 2001). Affective bias allows one to measure “positive” and “negative” emotional valences paired with specific stimuli; these are likely to map on to approach and avoidance behaviors, respectively (Berridge and Robinson, 1998). In the present study, we hypothesized that the affective bias toward sexual cues and the brain response to these cues would be positively correlated (i.e., a stronger brain response to sexual cues would correspond to a more positive bias towards sexual stimuli).

## Materials and Methods

### Participants

Participants (*n* = 60) were recruited from a federally-supported Title X (serving low-income individuals) family planning clinic and from a nearby university; both recruitment sites were located in a large urban area in the mid-Atlantic region of the United States. Flyers were posted and handed to participants by study team recruiters in the clinic waiting room and posted in the surrounding university campus. Potential participants expressed interest in the study by calling the phone number on the flyer or talking with recruiters in person. Eligibility screening was performed in a private location in the clinic or over the phone. The eligibility criteria included: women of ages 18–24 years who had vaginal sex (defined as penis in vagina) in the past 3 months, who were able to speak and read English at a 6th-grade level or above, and who were able to independently provide written informed consent. Exclusion criteria beyond standard fMRI contraindications (e.g., claustrophobia; metal in the body) included: pregnancy or plans to become pregnant in the next year or having given birth in the last 3 months, use of a copper IUD for birth control, being HIV-infected, having serious medical abnormalities (e.g., cardiovascular, neurological, endocrine, etc.) or untreated diabetes or hypertension, history of head trauma, history of seizure disorder, or currently under the influence of drugs or alcohol. Participation in any other studies was assessed and if these involved medications that might interfere with the fMRI, participants were excluded. Substance use was assessed by urine screens, recent alcohol use was assessed by breathalyzer, and pregnancy was assessed by a urine test prior to the fMRI session. Eligibility screening was supervised by an individual with a master of social work degree. After the fMRI session, the participant received compensation of $110 for the two-session visit. This study had Institutional Review Board approval and complied with the Declaration of Helsinki.

### Data Collection

Subjects participated in two sessions, typically scheduled on consecutive days. In the first session, participants completed informed consent, surveys, and an interview about sexual behaviors in the past 3 months using the Timeline Follow-Back (TLFB) method (Copersino et al., 2010). Information on other sensitive topics (e.g., intimate partner violence) was gathered using Audio Computer-Assisted Self-Interviewing (ACASI) that increases the accuracy of self-reported data (Newman et al., 2002). During the ACASI portion, participants wore headphones and listened as questions were read to them while also viewing the written questionnaire on a computer screen and entered responses on the computer. Participants were asked to provide demographic and health information by completing a paper survey.

#### Behavioral and Environmental Variables

Questions assessed for age, education (participant and mother’s), race, ethnicity, substance use, and sexual behavior history. Validated scales were used to measure impulsivity (Stanford et al., 2009), sensation seeking (Stephenson et al., 2007), risk-taking (Lejuez et al., 2002), anxious attachment (Kershaw et al., 2007), depression (Radloff, 1977), maltreatment (Bernstein et al., 2003), and intimate partner violence (IPV) (Garcia-Moreno et al., 2005).

#### STI/HIV Sexual Risk Behavior Measure

The primary measure for sexual risk was assessed by condom use during sex in the past 3 months. Participants who had sex with a condom 100% of the time in the past 3 months were considered the “Protected” group, and participants who had sex with a condom, less than 100% of the time in the past 3 months were considered the “Unprotected” group. To further define the Protected and Unprotected groups, a follow-up analysis incorporated the STIs/HIV risk of the participants’ partner. Participants who reported (or did not know) their partner had HIV, multiple partners, or an STI were considered “Risky Partners” (RP).

##### Other Sex-Related Behaviors

In addition to condom use, data were collected on other types of sex-related behaviors. These included: number of lifetime sexual partners, number of sexual partners in the past 3 months, anal sex since the age of 15, drug and alcohol use prior to sex, frequency of vaginal sex, and knowledge of partner’s STI and/or HIV status as well as partner’s sexual behaviors outside of their relationship (Centers for Disease Control and Prevention, 2018c).

#### fMRI Data Collection

In the second session, participants completed a blood-oxygen-level-dependent (BOLD) fMRI scan. The imaging center contains a Siemens 3 Tesla (Trio) research-dedicated magnet, an 8-channel head-coil, an LCD projector for stimulus presentation, air-conducting earphones, and a fiber optic response pad. Mirrors, attached to the head coil, are adjusted so that participants can focus attention on projected stimuli and instructions. Prior to the functional scans, a 3 min localizer scan and a T1-weighted high-resolution resting scan (5 min) were acquired. For functional scans: T2*-weighted BOLD images were obtained with a single-shot gradient echo-planar imaging sequence (field of view = 192 mm, matrix 64 × 64, TR = 2 s, TE = 30 ms, flip angle = 80°).

**Figure 1:**
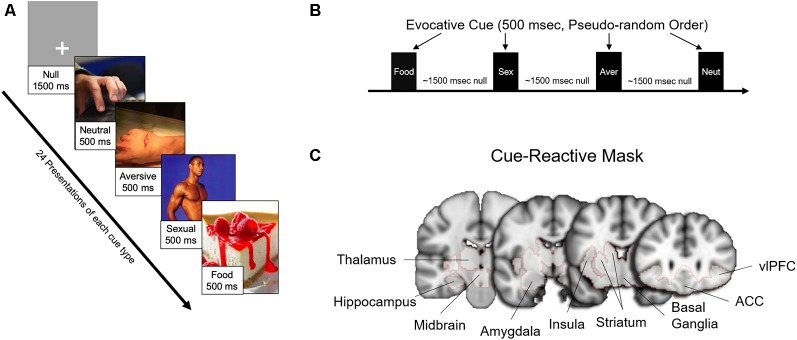
Task design, evocative cues, and cue-reactive mask. **(A)** Examples of evocative (sexual, aversive, food) and neutral cues. **(B)** Each cue was presented 24 times each for 500 ms in pseudorandom order interspersed with approximately an average of 1,500 ms (random duration between 1,000 and 2,000 ms) of a gray screen with crosshair (null). **(C)** Brain activity for the main findings was examined with a mesolimbic “cue-reactive” mask, with regions of interests based on previous studies of sexual stimuli and other evocative cues.

Fifty-four participants (of the 60 enrolled) completed the fMRI scanning session. Six subjects were unable to proceed with the fMRI visit [claustrophobia (*n* = 4), heart murmur (*n* = 1), dental retainer (*n* = 1)]. As in previous studies from our lab (Childress et al., 2008; Wetherill et al., 2014), the session included a “fast” event-related fMRI task with 24 novel 500 ms target cues in four categories [food (*n* = 24), sexual (*n* = 24), aversive (*n* = 24), and neutral (*n* = 24); Figure 1], from which usable data was gathered from 52 patients. More than half of the sexual cues and all of the aversive cues were selected from the top quartile (e.g., “most unpleasant” and “most pleasant,” respectively) of the International Affective Picture System (Lang et al., 1999). The remainder of the sexual cues were specifically generated to reflect the diversity of our sample. Target stimuli were interspersed with gray screens with a single crosshair presented at a random duration between 1,000 ms and 2,000 ms, an average of approximately 1,500 ms (Figure 1).

#### Affective Bias

After the fMRI scan, participants completed an off-scanner affective priming task, that determined the hedonic valence of visual sexual and condom cues by measuring the ability of these cues to influence (i.e., prime) the identification of nouns as positive (e.g., joy, paradise) or negative (e.g., murder, vomit). Images with positive valence (e.g., sexual cues) have been shown to facilitate the speed and accuracy for identifying positively-valenced nouns, and, conversely, to slow the reaction time of negatively-valenced nouns. Images with a negative valence (e.g., aversive) have the opposite effect (Olson and Fazio, 2001; Childress et al., 2008). A total of 12 sexual cues were chosen from a subset of those used on the scanner task (see below), and another 12 images of condoms were free-to-use images chosen from internet sites.

### Data Analysis

#### Demographic Health and Behavioral Data

Survey data were analyzed descriptively for frequency, mean, median and range. Demographic and sexual risk behavioral data were compared between sexual risk subgroups (Protected vs. Unprotected), using Chi-square and Fisher’s exact test for categorical variables, and *t*-tests for continuous variables. Health and behavioral data were analyzed with SPSS (IBM, 2016) and MATLAB (The MathWorks, 2019).

#### Imaging Data

Data processing was carried out in SPM12^1^ run under MATLAB R2019a. Each participants’ images were slice-timing corrected, realigned, co-registered to high-resolution to structural images, and subsequently normalized to MNI standard space and smoothed with the FWHM kernel of 9 mm. The motion statistics for each subject were examined to ensure that motion did not exceed 2 mm in any plane. For the first-level analysis, a canonical hemodynamic response function with time and dispersive derivatives was fitted to the onset of each event. The following contrasts were defined to assess the cue effect: sexual vs. neutral, aversive vs. neutral and food vs. neutral.

##### Mesolimbic “Cue-Reactive” Mask

For each contrast, independent *t*-tests were conducted between sexual risk groups (Protected vs. Unprotected). Primary analyses were limited to subcortical regions [e.g., caudate, putamen, insula, amygdala, hippocampus, caudal orbitofrontal cortex (e.g., ventrolateral prefrontal cortex, or vlPFC), thalamus] associated with neural responses to sexual stimuli (Mitricheva et al., 2019) and other evocative cues (Childress et al., 1999; Franklin et al., 2007; Noori et al., 2016; Regier et al., 2017). These regions were combined into a mesolimbic “cue-reactive” mask (Figure 1) using the Harvard-Oxford probabilistic anatomical atlas included with FMRIB Software Library (FSL). Clusters were considered significant at *p* < 0.005, cluster-corrected (*k* > 130) with Monte-Carlo simulations, using 3dClustsim included in the most recent AFNI software (Cox et al., 2017). Images were displayed with Mango (Multi-Image Analysis GUI) software^2^. Results are displayed both at cluster-corrected *p* < 0.01 and *p* < 0.005 to illustrate the spread of activation surrounding the peaks. Parameter estimates were extracted from nodes to explore differences between sexual risk subgroups—Protected (with and without RP), Unprotected (with and without RP)—and to examine relationships with sexual risk variables and bias scores.

#### Affective Bias

An affective bias score was calculated for those who correctly completed the task (at least 70% of the nouns correctly identified as positive or negative). For each image category, mean reaction time for positive word trials was subtracted from the mean reaction time for negative word trials to obtain the mean affective bias score. Thus, positive reaction time scores reflected a more positive affective bias, and negative reaction time scores reflected a more negative affective bias. Bias scores were compared between Protected vs. Unprotected groups (and RP subgroups) with *t*-tests. Bias scores were also used to examine the relationship with the brain response to sexual (-neutral) cues within the mesolimbic mask. As described above, clusters were considered significant at *p* < 0.005, cluster-corrected (*k* > 130) with Monte-Carlo simulations, and images were displayed with at both at cluster-corrected *p* < 0.01 and *p* < 0.005 to illustrate the spread of activation surrounding the peaks.

## Results

### Demographic and Health Variables

The average age of participants was 21. The population had a diverse racial/ethnic profile; participants self-identified as African American (67%), Caucasian (24%), Asian (7%), Hispanic/Latino (6%), and American Indian/Taino (4%). The majority of participants were students (59%) with an average of 12.4 highest grade completed. In the past 30 days, 67% used alcohol, 31% used marijuana, and 9% used cigarettes.

Thirty-three percent (*n* = 18) of the emerging adult women participants used condoms 100% of the time in the past 3 months (Protected) and 67% (*n* = 36) used condoms less than 100% of the time, 26 of whom (72%) did not use condoms at all, in the past 3 months (Unprotected). For the 52 participants that completed the 500 ms brain imaging task, there were no differences of demographic variables between the Protected (*n* = 17) and Unprotected (*n* = 35) groups (Table 1), and the Protected and Unprotected groups did not differ on impulsivity, sensation seeking, BART scores, or anxious attachment. Significantly more of the Unprotected group had been victims of intimate partner violence (80% vs. 47%, *χ*^2^ = 5.73, *p* < 0.05; Table 1), but they did not differ from the Protected group on childhood maltreatment scores.

**Table 1:** Demographics and health variables.

	Protected (*n* = 17)	Unprotected (*n* = 35)	*P*-value
Age	20.8	21.4	^1^*p* = 0.78
Mom finished high school	88%	77%	^2^*p* = 0.34
Recruitment site	47%	69%	^2^*p* = 0.13
Race:			
African American	41%	69%	^3^*p* = 0.09
Caucasian	24%	20%	
Asian/American Indian/Other	35%	11%	
Hispanic	24%	11%	^2^*p* = 0.26
Smokes cigarettes	0%	14%	^2^*p* = 0.11
Alcohol use	69%	66%	^2^*p* = 0.83
Cannabis use	38%	29%	^2^*p* = 0.52
Intimate Partner Violence	47%	80%	^2^*p* = 0.02
Impulsivity	60.5	60.5	^1^*p* = 0.99
Anxious attachment	7.6	7.9	^1^*p* = 0.74
Sensation seeking	24.8	24.3	^1^*p* = 0.75
Depression	20.2	15.9	^1^*p* = 0.21
BART	28.6	29.4	^1^*p* = 0.60
*Sexual risk (and other sex-related) variables*			
Number of lifetime partners	4.2	7.8	^1^*p* = 0.02
Multiple partners (past 3 months)	18%	23%	^2^*p* = 0.67
Anal sex since age 15	12%	51%	^2^*p* = 0.006
Alcohol use before sex	59%	37%	^2^*p* = 0.14
Drug use before sex	18%	6%	^2^*p* = 0.17
Frequency of sex (past 3 months)	5.3	17.3	^1^*p* = 0.01
Risky Partner	82%	43%	^2^*p* = 0.007
History of STI	24%	46%	^2^*p* = 0.12

*^1^Independent t-test; ^2^Chi-Squared test (2 × 2); ^3^Chi-Squared test (2 × 3)*.

Except for alcohol use before sex and partner status, measures related to sexual risk were generally higher in the Unprotected group (Table 1). Significant differences (FDR-corrected) were found for anal sex since the age of 15″ (51% vs. 12%, *χ*^2^ = 7.78, *p* < 0.01), total amount of lifetime sexual partners (7.8 vs. 4.4, *t*_(52)_ = 2.27, *p* < 0.05), and frequency of vaginal sex in the past 3 months (16.8 vs. 5.2, *t*_(52)_ = 2.79, p *=* < 0.01; Table 1). In contrast, significantly fewer women in the Unprotected group had “Risky Partners” (RP) in the past 3 months (43% vs. 82%, *p* < 0.01).

### Imaging

#### Brain Response to Evocative Cues

**Figure 2:**
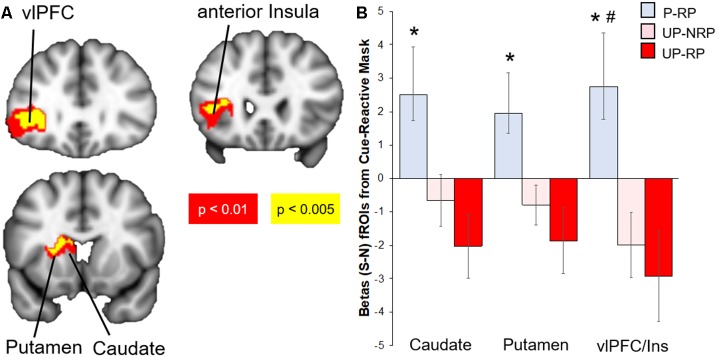
Differential brain response to sexual (S)–neutral (N) cues among sexual risk groups. **(A)** Protected > Unprotected groups: activity in the mesolimbic mask, with nodes in the putamen, caudate, anterior insula, and lateral OFC, was higher in the Protected vs. Unprotected group. **(B)** The Protected (P, blue) with risky partners (RP) group had the highest brain mesolimbic response that significantly differed from both the Unprotected-RP (UP-RP, orange) and Unprotected-NoRP (UP-NRP, red) groups in the vlPFC but only the Unprotected-RP group in the caudate and putamen. *Significant difference vs. Unprotected-RP group. ^**#**^Significant difference vs. Unprotected-NoRP group.

Compared to the Unprotected group, the brain response to sexual (-neutral) cues (controlling for IPV) in the Protected group was higher in the cue-reactive mask, with nodes in the dorsal striatum (caudate and putamen), anterior insula, and vlPFC [voxel-level threshold: *p* < 0.005, cluster-corrected (*k* > 130); Figure 2]. No significant results were found within the cue-reactive mask when comparing Protected vs. Unprotected on brain response to aversive or food cues. Whole-brain results are presented in Table 2 and displayed in the Supplementary Figure S1.

**Table 2:** Whole-brain results.

Region	Coordinates x, y, z	Peak *t*-Value	Number of Voxels
*Protected > Unprotected: Sexual Cues*			
vlPFC	−30, 34, −6	3.12	328
Fusiform gyrus	52, −38, −18	3.69	563
*Protected > Unprotected: Aversive Cues*			
Sup. front lobe	−20, 20, 60	4.7	920
Mid. front lobe	18, 26, 58	3.44	296
*Protected > Unprotected: Food Cues*			
Cerebellum	32, −58, −36	3.66	435
*Sexual Cues with Sexual Bias*			
dlPFC (left)	−40, 38, 24	4.07	437
dlPFC (right)	38, 34, 30	3.78	508
Putamen (right)	26, 16, 8	3.68	261
Inf. Par. Lobe	58, −30, 54	3.53	268

*Results at p < 0.005, cluster corrected (k > 256). vlPFC, ventrolateral prefrontal cortex; MTL, middle temporal lobe; sup front lobe, superior frontal lobe; mid front lobe, middle frontal lobe; dlPFC, dorsolateral prefrontal cortex; inf par lobe, inferior parietal lobe*.

##### Sexual Cues Response by Sexual Risk Subgroups

Because the brain response to sexual cues may have differed due to differences in partner status [see “Materials and Methods” and “Results” section above; the majority of the Protected group (15/18) had a “risky partner” (RP), while less than half of the Unprotected group (16/36) had an RP], we investigated groups by sexual risk subgroups (Protected-RP, *n* = 15; Unprotected-NoRP, *n* = 20; and Unprotected-RP, *n* = 16; only three of the women in the Protected group did not have RP and thus were excluded from the analysis, due to the small number). Extracted parameter estimates from significant clusters (caudate, putamen, and vlPFC) were compared between three subgroups. The results (FDR-corrected) showed that the Protected-RP group had greater brain response to sexual cues in the vlPFC compared to both the Unprotected-NoRP (*t*_(32)_ = 2.67, *p* < 0.05) and Unprotected-RP groups (*t*_(27)_ = 2.71, *p* < 0.05). In addition, the results show that the Protected-RP group had greater brain response to sexual cues in the caudate (*t*_(27)_ = 2.67) and putamen (*p* < 0.05; *t*_(27)_ = 2.48, *p* < 0.05) compared to the Unprotected-RP group, while the difference of brain response between the Protected-RP and Unprotected-*NoRP* group trended towards significance in the caudate (*p* = 0.067) and putamen (*p* = 0.058). The brain response between the Unprotected (NoRP vs. RP) groups did not differ (Figure 2).

##### Sexual Cue Response: Correlation With Sex Frequency

**Figure 3:**
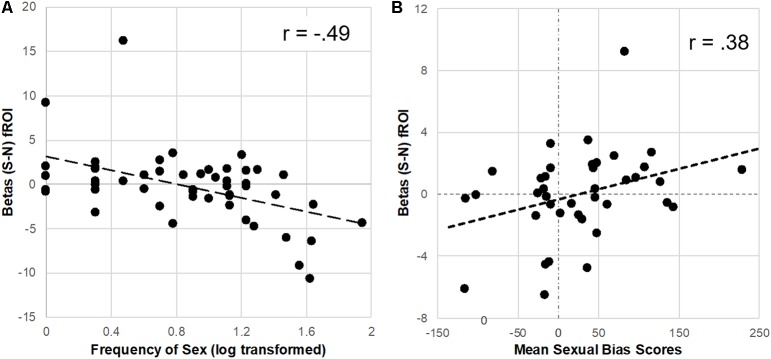
Parameter estimates (betas) from the Protected > Unprotected [sexual (S)–neutral (N) cues] differential brain response correlated with the frequency of sex and affective sexual bias scores. **(A)** Neural responses in the dorsal striatum (fROI) to sex (-neutral) cues were inversely correlated with the frequency of sex in the past 3 months. **(B)** In contrast, neural responses in the putamen to sexual (-neutral) cues were positively correlated with affective sexual bias scores.

To further explore the reduction of brain response to sexual cues observed in the Unprotected (vs. Protected) group, extracted parameter estimates were correlated with the frequency of sex in the past 3 months. The distribution of the sex frequency variable was not gaussian but instead positively skewed, thus sex frequency was log-transformed prior to analyses and plotting (Manikandan, 2010). There was a significant inverse correlation (FDR-corrected) of frequency of sex in past 3 months and the brain response to sexual (-neutral) cues in the caudate (*r* = −0.47, *p* < 0.01), putamen (*r* = −0.49, *p* < 0.01), and vlPFC (*r* = −0.47, *p* < 0.01; Figure 3).

### Affective Bias

#### Sexual Cue Response: Correlation With Sexual Bias

For the subset of participants (*n* = 39) who correctly completed the affective bias test (achieved at least 70% accuracy), extracted parameter estimates from the difference between Protected > Unprotected groups brain response to sexual (-neutral) cues (see “Results” section above) were used to test the relationship with implicit affective bias to sexual cues. Results showed a significant positive correlation (FDR-corrected) with parameter estimates from the putamen (*r* = 0.38, *p* < 0.05; Figure 3) but only a trend was found with dorsal caudate parameter estimates (*r* = 0.28, *p* = 0.09), and no significant relationship was found with vlPFC parameter estimates.

#### Affective Bias Scores

**Figure 4:**
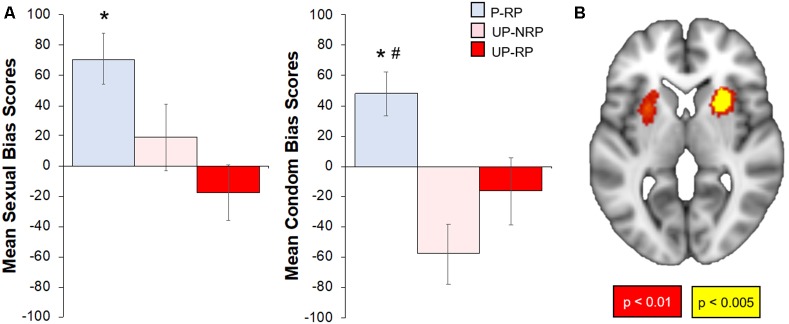
Differences in affective sexual bias scores by sexual risk group; correlation of affective sexual bias scores with the brain response to sexual (-neutral) cues. **(A)** Overall, the Protected group had higher affective bias scores to sexual and condom cues. When accounting for RP, the Protected RP (P-RP, blue) group had significantly higher sexual bias scores than the Unprotected RP (UP-RP, red) group but not the Unprotected-NoRP (UP-RP, orange) group; the P-RP group had higher condom bias scores than both Unprotected (RP and NoRP) groups, but bias (sexual or condom) scores did not differ between UP groups. **(B)** Sexual bias scores positively correlated with regions within the cue-reactive mask, including nodes in the bilateral putamen (*p* < 0.01–0.005, cluster corrected). *Significant difference vs. Unprotected-RP group. ^#^Significant difference vs. Unprotected-NoRP group.

The Protected group had a positive bias to both sexual and condom cues, while the Unprotected group had significantly lower bias (vs. the Protected group) scores to sexual (*t*_(37)_ = 3.71, *p* < 0.05) and condom cues (*t*_(37)_, *p* < 0.01). To check whether bias scores differed by the risk of the sexual partner (RP variable), we examined bias scores in subgroups of Protected and Unprotected groups (see “Materials and Methods” and “Results” section; Figure 4). The Protected-RP group had higher affective bias scores to sexual cues compared to the Unprotected-RP group (*t*_(20)_ = 3.54, *p* < 0.01) but not the Unprotected-*NoRP* group (*t*_(24)_ = 1.82, *p* = 0.11). There were no differences between Unprotected (NoRP vs. RP) groups. The Protected-RP group had higher (FDR-corrected) condom bias scores compared to both the Unprotected-RP group (*t*_(20)_ = 2.52, *p* < 0.05) and the Unprotected-NoRP group (*t*_(24)_ = 4.21, *p* < 0.01). Again, there were no differences between Unprotected (NoRP vs. RP) groups.

#### Exploratory: Sexual Bias and Brain Response to Sexual Cues

For the subset of 39 participants who successfully completed the affective bias task and the 500 ms brief cue fMRI task, the brain response to sexual (-neutral) cues were correlated with sexual bias scores. Results showed a significant positive relationship within the cue-reactive mask, with nodes centered in the bilateral putamen (Figure 4). Whole-brain results are presented in Table 2 and displayed in the Supplementary Figure S2.

## Discussion

In this study, emerging adult women (ages 18–24) who had sex in the past 3 months were divided into two groups: individuals who used a condom 100% of the time (Protected group) and individuals who did not use a condom 100% of the time (Unprotected group). These two groups were compared on their brain response to evocative [sexual, food, aversive (vs. neutral) cues] in several mesolimbic regions (Figure 1). Based on prior studies, primarily in males, it was expected that heightened mesolimbic response to sexual cues would correspond with higher sexual risk (i.e., unprotected sex). However, in the present study, it was instead women in the “Protected” (vs. Unprotected) group who exhibited a heightened mesolimbic brain response to sexual cues (Figure 2). Because the Protected group had more “risky” partners (RP, i.e., reported or did not know that their partner had HIV, STIs, and/or other sexual partners), follow-up analyses were conducted to account for partner status. Compared to both the Unprotected (NoRP and RP) groups, generally, the Protected-RP group had more activation in nodes within the cue-reactive mask (Figure 2). Even though there was a tendency of the Protected-RP group to have the highest response and Unprotected-RP to have the lowest response, there were no differences between the Unprotected (NoRP vs. RP) groups. Interestingly, activation nodes within the mesolimbic mask were inversely correlated with the frequency of sex in the past 3 months (Figure 3). In other words, the more sex individuals reported from the past 3 months, the lower the mesolimbic response to sexual cues. Finally, in a subset of participants who successfully completed an affective bias task, the Protected (vs. Unprotected) group showed a higher positive affective bias for sexual and condom cues. In line with our secondary hypothesis, sexual bias scores were positively correlated with the mesolimbic response to sexual cues, with nodes in the bilateral putamen (Figures 3, 4). In other words, it was expected that sexual bias scores would have a positive relationship with the mesolimbic response to sexual cues, however, it was unexpected that higher sexual bias scores and correlation with an increased brain response to sexual cues was higher in the group at *lower* risk for STIs/HIV.

Our hypotheses were based on prior literature generally reporting greater reward circuit activation for those with higher sexual risk behaviors; however, most of the emerging studies on the relationship of brain response to sexual cues and sexual risk behaviors have thus far been in males (Voon et al., 2014; Seok and Sohn, 2015). Previous literature indicates that there is a difference between male and female attitudes about condom use. For example, males have reported that condom use is associated with a lack of pleasure, whereas females have reported that condom use by their partner is associated with protection from negative consequences (Martinez-Donate et al., 2004; Hill et al., 2011; Calsyn et al., 2013). Our findings suggest that sexual associations at the level of the brain and behavior (affective biases) are more favorable for women whose partners use condoms. While some studies have reported male vs. female differences of neurobiological and behavioral responses to sexual stimuli (Rupp and Wallen, 2008; Hill et al., 2011), a recent meta-analysis found that females and males generally activate the same brain regions in response to sexual cues (Mitricheva et al., 2019). In addition, while a recent study found that differential patterns in the subcortical response to *non-sexual* cues between males vs. females were predictive of sexual risk behaviors (Victor et al., 2015), it is unclear whether there would be differences in the mesolimbic response to *sexual* cues between males vs. females with varying degrees of STIs/HIV sexual risk behaviors.

Given the previous literature and present study, one interpretation of our results might involve a level of safety, in that women may feel more protected, worry less about the negative consequences, and may, therefore, enjoy sex more when their partners use condoms. Though not mutually exclusive, another interpretation may be that the Protected group represents the standard response (heightened reward activity and positive bias to sexual stimuli), while the Unprotected group, particularly the Unprotected-RP subgroup, might represent an atypical response (diminished reward activity and sexual bias to sexual stimuli). Given that the Unprotected-RP subgroup reported significantly more negative experiences with sexual partners, such as a history of STIs and physical IPV, compared to the other subgroups (uncorrected; Protected-RP: *p* = 0.04, *p* = 0.01, respectively; Unprotected-NoRP: *p* = 0.03, *p* = 0.03, respectively), that may partially explain the decreased reward response to sexual stimuli, potentially exacerbated by more sexual encounters (i.e., frequency of sex). Though our numbers were too few to examine the interaction of all these variables, future studies would provide further elucidation.

Significant nodes within the cue-reactive mask (e.g., dorsal striatum, insula) that differed between the Protected and Unprotected groups have been shown to be involved in the processing of visual sexual stimuli (Mitricheva et al., 2019). The caudate and putamen process both positive and negative stimuli (Lammel et al., 2014), driving reward-seeking behaviors and motivational states (Wise, 2004), such as pleasurable eating (e.g., Small et al., 2003), drug craving (e.g., Breiter et al., 1997; Wong et al., 2006; Volkow et al., 2006), sexual-related activities (see review, Gola and Draps, 2018), and the pursuit and loss of monetary value (e.g., Knutson et al., 2000). However, they have been found to differ in other processes, such as those associated with deliberative (caudate) and habit-based (putamen) behaviors (e.g., Yin and Knowlton, 2006; Graybiel, 2008; Regier et al., 2015). In the present study, positive sexual bias associated with activation to sexual cues may indicate an increased reward response in the Protected group, whereas the decreased response in the Unprotected group may indicate an attenuated reward response to sexual stimuli. This attenuated reward response may be particularly relevant to the Unprotected-RP group, which on average had negative sexual bias scores and lower striatal responses to sexual cues. Other brain responses that differed between groups included nodes primarily in the vlPFC but that also overlapped with the anterior insula. The vlPFC receives projections from dopaminergic regions and has been implicated in reward-related decisions (Sakagami and Pan, 2007; Treadway et al., 2012) and interoceptive signals related to the processing of evocative stimuli (Seo et al., 2014). The anterior insula has also been shown to process visceral experiences (e.g., increased heart rate, nervous stomach) associated with evocative stimuli (Craig, 2009), and has recently been posited as a hub of appetitive motivational systems in risky reward-seeking behaviors (Naqvi and Bechara, 2009). Therefore, abnormally low activity in the insula and vlPFC, as observed in the Unprotected group, may be associated with more risky behavior.

The challenges of the current study may be used to stimulate and guide future research. For example, although differences between groups were found for affective bias scores of condoms, condom images were not included in the fMRI imaging design. Results from the present study may imply sex differences of the brain response to sexual cues between females and males at higher and lower risk for STIs/HIV; however, a future study explicitly testing these apparent differences, within male and female cohorts tested in similar paradigms, would be highly informative. It is notable that there were potential differences in condom use practices by race/ethnicity with proportionally more Hispanic and Asian/Pacific Islander and fewer African-American women in the protected group. Given the multiple factors underlying race/ethnicity categories, this is an interesting result that merits further considering in future research, employing larger samples allowing for disaggregation of these categories. Though the current study featured passive exposure to brief evocative cues, the results encourage future studies with tasks that can explicitly probe decision-making systems (e.g., reinforcement learning) utilizing these evocative cues. Additional tasks, parameters, or even different analyses might reveal brain structures (e.g., ventral striatum, amygdala, hippocampus) undetected by the current study. Finally, although the current study has an adequate sample size (*n* = 52) for examining the brain response to sexual stimuli associated with sexual risk behaviors in the overall group (even) larger future sample sizes would enable examination of other heterogeneities, as mentioned above, as well as others (e.g., mood and anxiety disorders) that may both impact the brain response to evocative cues and sexual risk behaviors.

## Conclusions

Individuals at higher risk for STIs/HIV had lower activation in subcortical areas in response to sexual cues; they had a less positive affective bias to sexual cues and condoms compared to individuals at lower risk for STIs/HIV, and the bias to sexual cues was positively correlated with the subcortical brain response to sexual cues. Together, these results indicate that women whose partners use condoms may have a higher reward response to sexual cues, or that the women whose partners did not use condoms may have an attenuated reward response to sexual cues. Understanding the relationship of brain response to appetitive cues associated with greater sexual risk can help to inform treatment interventions that target these brain responses with behavioral therapy, medication, or both. In addition, the availability of medications for pre-exposure prophylaxis (PrEP) to prevent HIV infection (Flash et al., 2014) has energized research efforts toward identifying individuals at increased STI/HIV risk.

## Data Availability Statement

The datasets generated for this study are available on request to the corresponding author.

## Ethics Statement

The studies involving human participants were reviewed and approved by Institutional Review Board. The patients/participants provided their written informed consent to participate in this study.

## Author Contributions

AT, AC, and PR contributed to the conception and design of the study. ZM acquired fMRI data. KJ, ZM, and CM performed initial analyses. PR performed all other statistical analyses and wrote first draft of the manuscript. All authors contributed to manuscript revision and read and approved the submitted version.

## Conflict of Interest

The authors declare that the research was conducted in the absence of any commercial or financial relationships that could be construed as a potential conflict of interest.

## Footnotes

^1^http://www.fil.ion.ucl.ac.uk/spm

^2^http://ric.uthscsa.edu/mango/mango.html
