# Supplementary figures and images for: Women at Greater Sexual Risk for STIs/HIV Have a Lower Mesolimbic and Affective Bias Response to Sexual Stimuli
Source: Front Behav Neurosci. 2020 Jan 10;13:279. doi: 10.3389/fnbeh.2019.00279 (PMC6965060; doi:10.3389/fnbeh.2019.00279)

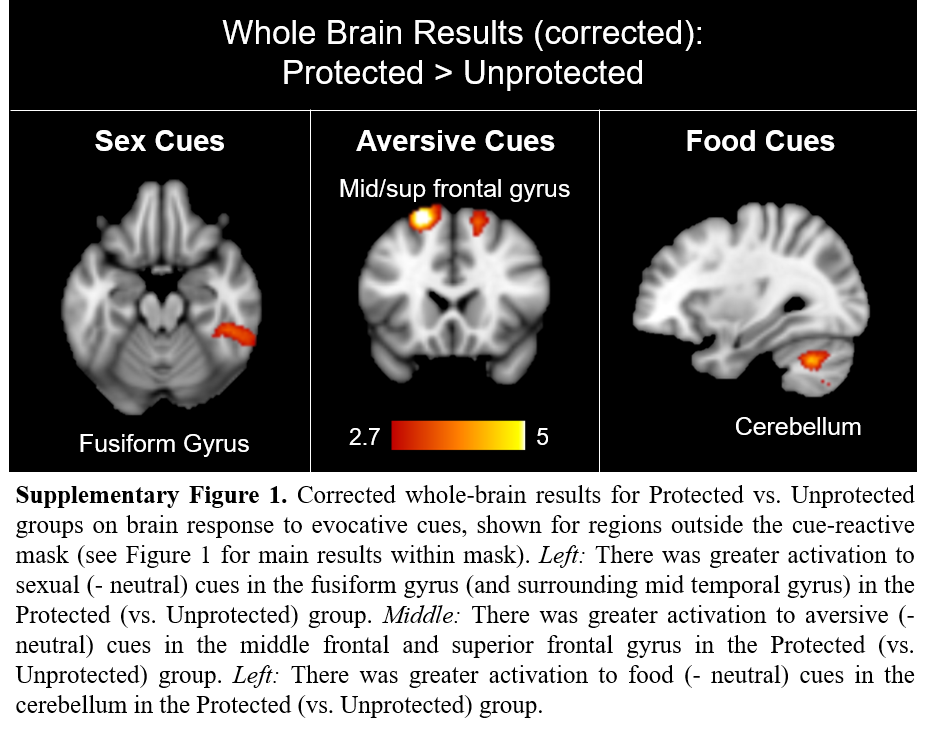

Supplement: Supplementary file 1 [file Image_1.TIF]

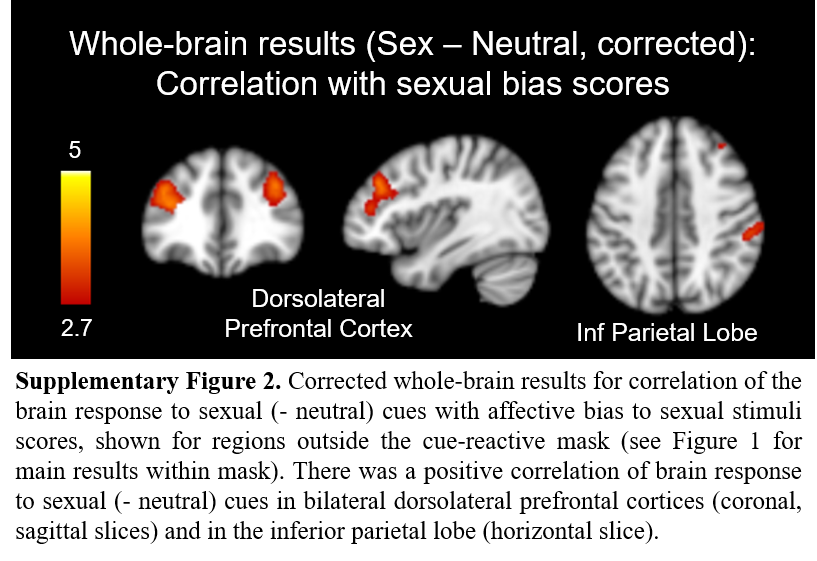

Supplement: Supplementary file 2 [file Image_2.TIF]
